# Potential roles of *FAT1* somatic mutation in progression of head and neck cancer

**DOI:** 10.18632/oncoscience.558

**Published:** 2022-05-12

**Authors:** Zhuo G. Chen, Yong Teng

**Affiliations:** ^1^Department of Hematology and Medical Oncology, Winship Cancer Institute, Emory University School of Medicine, Atlanta, GA, USA

**Keywords:** FAT1, head and neck cancer, somatic mutation, genetic and proteomic analysis, survival benefits

Head and neck squamous cell carcinoma (HNSCC) is the sixth most common cancer worldwide [1]. It disturbs patients’ vital upper aero-digestive function. Treatment outcomes for patients with HNSCC remain poor in past decades due to the lack of effective therapeutic options, thereby, discovery and evaluation of new medications are of tremendous importance for improving patients’ survival. Human papillomavirus (HPV) status remains one of strong indicators of survival, however, HPV-unrelated disease carrying a 5-year overall survival (OS) rate of less than 50% [2]. The challenges in effectively treating HNSCC are attributed to its extreme heterogeneity as far as anatomic locations and genetic aberrations. Major gaps in understanding the biology of disease continue to be the main reason behind the paucity of effective therapeutic interventions.

*FAT1* encodes a member of the cadherin-like protein family. Under normal physiological conditions, FAT1 serves as a molecular “brake” on mitochondrial respiration [3] and acts as a receptor for a signaling pathway regulating cell-cell contact interaction and planar cell polarity [4, 5]. Loss of *fat* leads to cell cycle dysregulation and hyperproliferation in *Drosophila* larval imaginal discs [6]. Recently, *FAT1* mutations were identified in human cancers and may contribute to Wnt activation, suggesting that FAT1 may serve as a tumor suppressor in human cells [7]. The *FAT1* mutant was found to inactivate the Hippo regulatory complex, which leads to activation of YAP1 in HNSCC as reported by Martin et al. They also indicated that the *FAT1* gene alteration rate was as high as 29.8% in HNSCC, which is the highest among solid tumors [8]. *FAT1* mutation was reported to be more common in HPV-negative (HPV−) than in HPV-positive (HPV+) HNSCC (28% vs. 2.8%). Mann et al., examined 16 HNSCC cell lines and reported a *FAT1* mutation rate of 43% [9]. One recent study on HNSCC patients in Taiwan reported a 29% mutation rate of *FAT1* and showed significant correlations of *FAT1* mutations with lymph node status and worse disease-free survival (DFS) [10]. However, the effect of *FAT1* mutation on HNSCC malignant phenotypes has not been extensively investigated, and little is known about its clinical implications.

**Figure 1:**
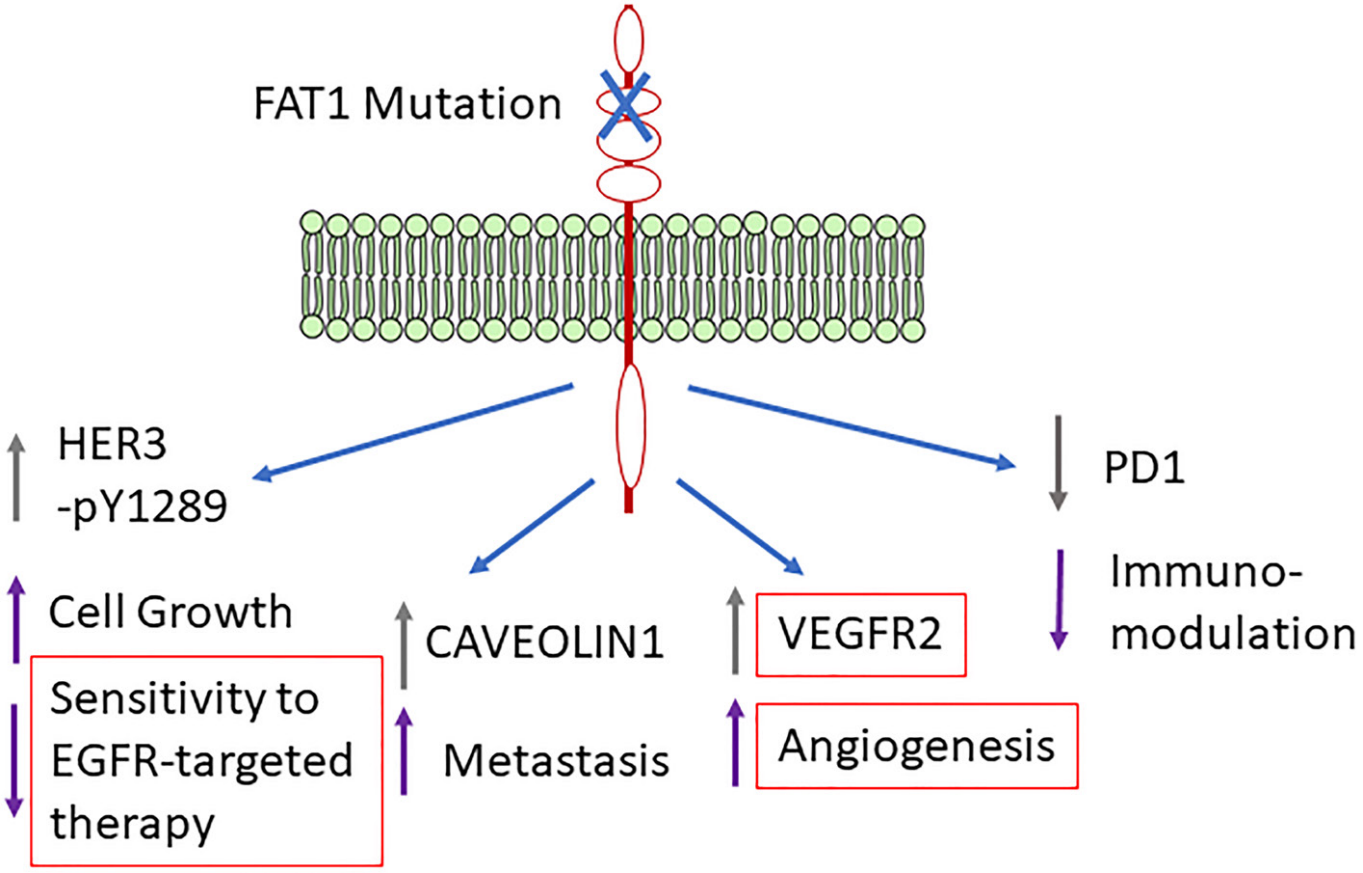
Potential altered cellular functions mediated by mutated *FAT1* associated molecules.

This manuscript (Chen et al., The Proteomic Landscape of Growth Factor Signaling Network Associated with FAT1 Mutations in Head and Neck Cancer. Cancer Res 2021; 81:4402–16) is the first comprehensive proteomic analysis identifying altered protein expression and activation status associated with FAT1 mutation in HNSCC using the Reverse Phase Protein Arrays (RPPA) dataset from TCGA project. This platform represents a powerful approach with globally functional proteomics, including more than 200 validated antibodies detecting proteins or phosphorylation sites indicating activation status (Cancer Proteome Atlas, TCPA, http://tcpaportal.org). The selection of this panel of antibodies was based on antigens representing molecules with the most critical cancer biology implications, such as growth factors, signaling molecules, transcription factors, cell cycle, and apoptosis modulators. This study used the TCGA genomic, expression, and RPPA datasets to compare differentially expressed proteins and their phosphorylated sites in HNSCC tissues associated with FAT1 mutation status. A panel of differentially expressed proteins, stratified by FAT1 mutation status, were identified to be involved in the activation of growth factors and signaling pathways with potential effect on cancer cell proliferation, metastasis, angiogenesis, and immunomodulation (Figure 1).

The most significant and novel finding is the identification of a broader proteomic landscape of predominant oncogenes and tumor suppressors and potentially druggable targets associated with mutant or wild-type *FAT1*. It was observed increased expression of a network of proteins that includes cell surface receptors, such as HER3_pY1289, VEGFR2, and PDL1, plus IGFR signaling mediator IRS1, and cell cycle modulator CMYC, in more than 90 HNSCC patient samples with *FAT1* mutation. Although the highly mutated *FAT1* molecule itself has not been identified as a therapeutic target in HNSCC, due to its nature as a tumor suppressor, these surface receptors and signaling molecules associated with *FAT1* mutation are well-investigated therapeutic targets with available targeted agents and possible links to immune-related pathways. Specifically, this study revealed that in both total and HPV(−) patients, HER3_pY1289 was upregulated in *FAT1* mutated HNSCC. HER3 is one of the EGFR family members and HER3_pY1289 is the activated form that transduces signaling after partnering with other EGFR family members. It was also observed that *FAT1* knockout could impair EGFR phosphorylation/activation in HNSCC cell lines, indicating the potential impact of FAT1 in EGFR signaling. In addition, IRS1, a key regulator of IGF-1R, is also upregulated in *FAT1* mutated HNSCC. Crosstalk between HER3 and IGF-R1 signals could synergistically activate ERK/MAPK, PI3K/AKT, and RAS/RAF pathways and promote cell proliferation/survival, protein synthesis, and cell cycle through CMYC. Among other associated proteins, CAVEOLIN can alter cell shapes, and together with VEGFR2, enhance cell angiogenesis, stemness, migration, and invasion potential, which is consistent with the observations in skin and lung cancer models [11]. Furthermore, PD1 that mediates the immune escape of tumor cells is downregulated in the tumor microenvironment with mutant *FAT1*, leading to tumor immunotolerance.

Two significant etiologies of tobacco usage and HPV infection represent two major types of diseases, which activate distinct biological pathways in carcinogenesis. This study confirmed that the *FAT1* mutation rate was significantly higher in HPV(−) than HPV(+) patients in this cohort, suggesting *FAT1* mutation may play more critical roles in HPV(−) than in HPV(+) HNSCC. One clinical observation of the impact of mutant *FAT1* on PFS is supported by a previous study on HNSCC patients in Taiwan, which reported a significant correlation of *FAT1* mutation with worse DFS, but not OS [10].

In summary, these novel findings from proteomic analysis associated with *FAT1* mutation provide essential information as to potential biomarkers linked to *FAT1* and deserve future validation in HNSCC. The observations of survival benefits may directly impact the future development of *FAT1*-related prognostic variables and treatment strategies.
